# Inhibition of protein aggregation by zwitterionic polymer-based core-shell nanogels

**DOI:** 10.1038/srep45777

**Published:** 2017-04-04

**Authors:** Robin Rajan, Kazuaki Matsumura

**Affiliations:** 1 School of Materials Science, Japan Advanced Institute of Science and Technology, 1-1 Asahidai, Nomi, Ishikawa, 923-1292, Japan

## Abstract

Protein aggregation is a process by which misfolded proteins polymerizes into aggregates and forms fibrous structures with a β-sheet conformation, known as amyloids. It is an undesired outcome, as it not only causes numerous neurodegenerative diseases, but is also a major deterrent in the development of protein biopharmaceuticals. Here, we report a rational design for the synthesis of novel zwitterionic polymer-based core-shell nanogels via controlled radical polymerization. Nanogels with different sizes and functionalities in the core and shell were prepared. The nanogels exhibit remarkable efficiency in the protection of lysozyme against aggregation. Addition of nanogels suppresses the formation of toxic fibrils and also enables lysozyme to retain its enzymatic activity. Increasing the molecular weight and degree of hydrophobicity markedly increases its overall efficiency. Investigation of higher order structures revealed that lysozyme when heated without any additive loses its secondary structure and transforms into a random coil conformation. In contrast, presence of nanogels facilitates the retention of higher order structures by acting as molecular chaperones, thereby reducing molecular collisions. The present study is the first to show that it is possible to design zwitterionic nanogels using appropriate polymerization techniques that will protect proteins under conditions of extreme stress and inhibit aggregation.

Protein aggregation is a serious problem in the growing field of protein biopharmaceuticals^1^ and has hampered the development of protein-based biological formulations. Protein biopharmaceuticals have many advantages over other drugs; for example, they are highly specific, well-tolerated by the body, and eliminate the need for gene therapy^2^. However, formation of aggregates during drug administration causes proteins to lose their activity over time and may also trigger potentially dangerous immune responses^3^. In addition, the deposition of amyloid like fibrils has been associated with various neurodegenerative diseases such as Alzheimer’s and Parkinson’s diseases^4^,^5^.

Many compounds such as arginine^6^, proline^7^, osmolytes^8^, and quinones^9^ have been reported to suppress protein aggregation; however, excessively large amounts of these reagents are required, which limits their widespread application. Moreover, in general, these compounds are structurally simple and have limited potential for modifications. On the other hand, polymeric compounds are particularly promising in this regard owing to their inherent nature, as many parameters such as molecular weight, functionality, shape/architecture, etc. can be easily adjusted to increase their efficiency and meet clinical requirements. However, there are surprisingly few reports on the application of polymers in this area. In our previous study, we reported the synthesis of a polymer with very high activity against protein aggregation^10^; however, its efficiency was not satisfactory. The group of Akiyoshi fabricated nanogels by the self-assembly of cholesterol-bearing pullulan. These nanogels acted as molecular chaperones and had the ability to immobilize enzymes, protecting them against denaturation^11^,^12^,^13^.

Nanogels are nanosized hydrogels formed by hydrophobic, electrostatic, covalent, and coordinate bonds^14^. Their small size results in large and tuneable surface areas, which allow for easy encapsulation of molecules. They have numerous advantages including high biocompatibility and good permeation ability^15^, and have been widely applied in various fields such as diagnostics^16^, protein delivery^17^, and sensing^18^. In a recent study, Kotsuchibashi and Narain reported the design of dual temperature- and pH-responsive core-shell nanogels based on ethylene glycol, without the use of surfactants^19^.

Polyampholytes and zwitterionic polymers bear both positive and negative charges, and show unique properties such as cryoprotection of cells^20^,^21^,^22^,^23^,^24^. One interesting feature of zwitterionic polymers is its anti-biofouling property^25^,^26^. This is due to the presence of a nanoscale homogenous mixture of both the charges located close to each other^27^. As a result, neutralization of net charge in zwitterionic polymers takes place, resulting in diminution of ionic hydration, which causes water molecules in the vicinity to be less oriented (similar to that of bulk water). This property of zwitterionic polymers may be strongly related to their suppression of non-specific binding of proteins^28^. Moreover, zwitterionic polymers do not alter the structure of the hydrogen-bonded network of water molecules at the polymer-material interface^29^. Here, we present the synthesis of zwitterionic polymer-based nanogels via reversible addition-fragmentation chain transfer (RAFT) polymerization. A library of nanogels with shells of different molecular weights and cores containing various amounts of hydrophobic monomers was prepared, and their ability to suppress the thermal aggregation of lysozyme was investigated.

## Results and Discussion

### Synthesis and Characterization

**Figure 1:**
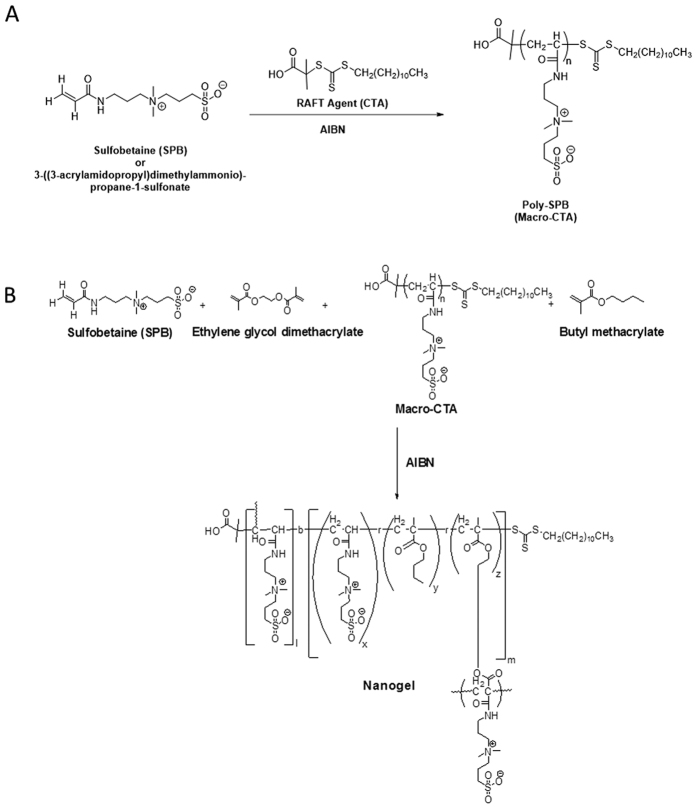
Synthesis of (**A**) Poly-SPB and (**B**) core-shell nanogel with BuMA in the core.

**Figure 2:**
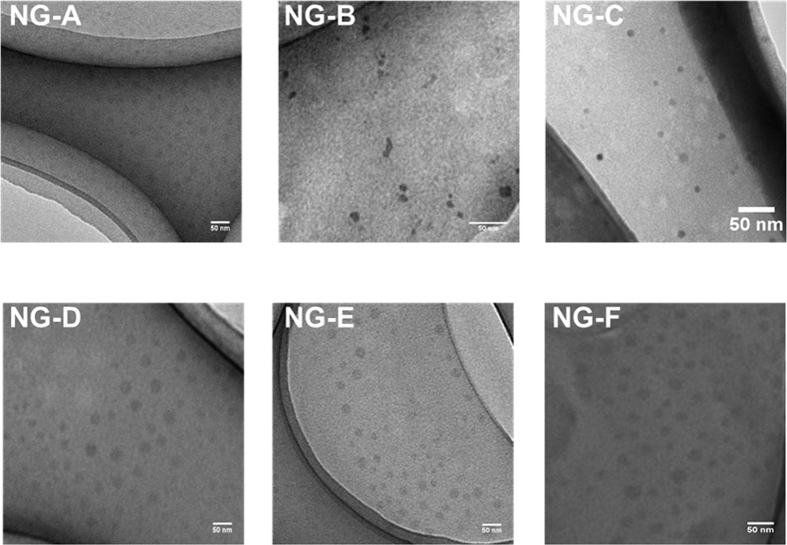
TEM images of nanogels. Scale bars represent 50 nm.

The nanogels were prepared by a two-step reaction. In the first step, macro-chain transfer agents (macro-CTAs) of zwitterionic monomer 3-((3-acrylamidopropyl)-dimethylammonio)-propane-1-sulfonate were synthesized by RAFT polymerization (Fig. 1A). The polymers were characterized by NMR (Figure S1) and gel permeation chromatography (GPC). The kinetic study of the macro-CTAs showed a linear conversion rate, thus validating the livingness of the polymerization reaction (Figure S2). GPC curves revealed a unimodal distribution with PDI values well within the range of living polymerization. The molecular weights were also well controlled. The core-shell nanogels were prepared using macro-CTA as a new chain transfer agent and including various amounts of hydrophobic monomer, namely butyl methacrylate (BuMA), in the core (Fig. 1B; Table 1). The nanogels were characterized using transmission electron microscopy (TEM) (Fig. 2) and dynamic light scattering (DLS) (Figure S3), which showed structures with spherical shape and size between 10 and 25 nm. The size of the nanogels increased with increasing molecular weight of the macro-CTA. This can be explained on the basis that macro-CTA constitutes the shell of the nanogel; thus, a higher molecular weight corresponds to a longer polymer chain in the shell, which leads to an overall increase of the nanogel size. Moreover, an increase in hydrophobicity leads to a more compact arrangement of the core of the nanogel and therefore to a decrease in size.

### Residual activity of lysozyme after heat treatment

**Figure 3:**
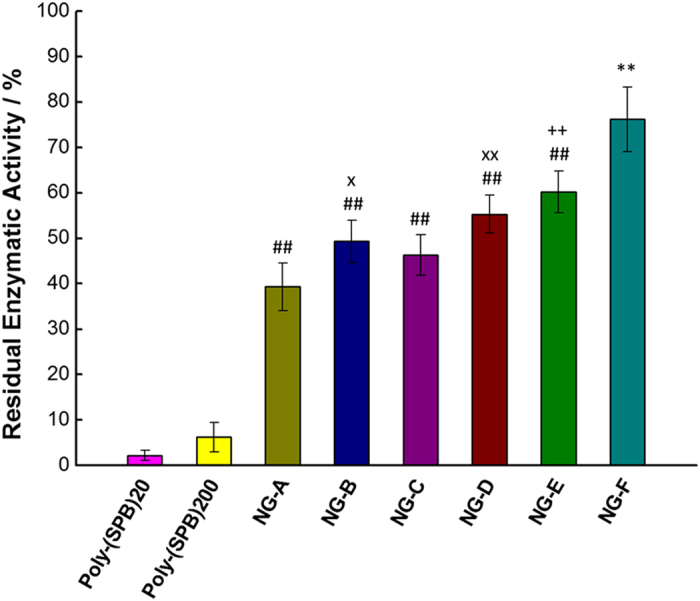
Enzymatic activity of lysozyme after treatment at 90 °C in the presence of different nanogels at 5% (w/v) concentration. Data are expressed as the mean ± SD of three independent experiments. **P < 0.01 vs. all other reagents; ^##^p < 0.01 vs. poly-(SPB)_20_ and poly-(SPB)_200_; ^++^p < 0.01 vs. NG-B; ^x^p < 0.05 vs. NG-A; ^xx^p < 0.01 vs. NG-A.

In order to evaluate the ability of the prepared nanogels to inhibit the thermal aggregation of lysozyme, the residual enzymatic activity of lysozyme after heat treatment, with and without additives, was measured using *Micrococcus lysodeikticus* cells^30^. We monitored the reduction in turbidity of the cell suspension due to lysozyme activity, which causes degradation of the bacterial cell wall and a dramatic decrease in absorbance at 600 nm. As shown in Fig. 3, all nanogels show very high efficiency. This may be attributed to the ability of the nanogels to protect the lysozyme from intermolecular interactions (due to their anti-biofouling property) between hydrophobic domains, which are responsible for denaturation. They are much more efficient than their linear analogues, possibly owing to their smaller size, which leads to greater shielding from intermolecular interactions. Comparison with our previous results reveals that even a twofold increase in polymer concentration (10%) did not produce a similar efficiency^10^. An increase in the amount of hydrophobic monomer or in the concentration of the nanogel also led to a higher efficiency (Figure S4), which can be explained by the tendency of hydrophobic species to cover the hydrophobic surfaces of proteins^31^. A similar trend was observed by increasing the molecular weight of the macro-CTA. It is known that increasing the molecular weight of zwitterionic polymers results in reduced protein adsorption^27^. Short polymer chains show anti-biofouling properties because of the formation of a hydration layer due to their hydrophilicity. In contrast, in longer polymer chains, the steric repulsions (for flexible polymer chains) in addition to the formation of a hydration layer results in a higher anti-biofouling activity^32^.

### Fibril formation by lysozyme

**Figure 4:**
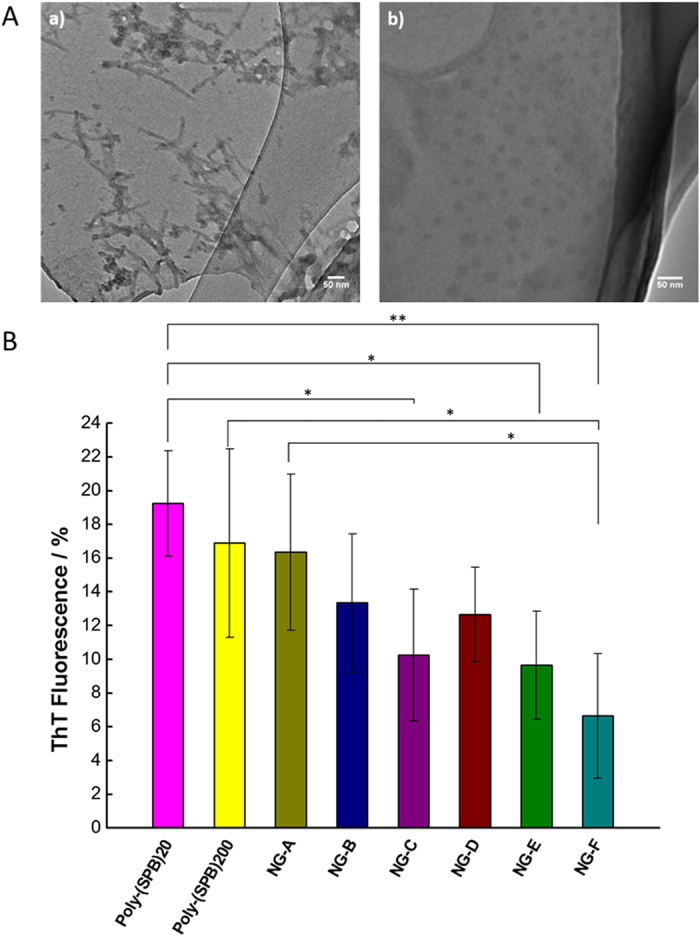
(**A**) TEM images of lysozyme solution after heat treatment (a) in PBS alone, and (b) with NG-F. Scale bars represent 50 nm. (**B**) Thioflavin T fluorescence of lysozyme (0.5 mg/mL) when heated to 90 °C for 30 minutes in the presence of different nanogels at 5% concentration (w/v %). Data are expressed as the mean ± SD of 3 independent experiments. *p < 0.05; **p < 0.01.

It is well established that amyloid protein fibrillation is associated with many neurodegenerative diseases, and its prevention is critically important for the development of protein biopharmaceuticals. We investigated the effect of nanogels on protein fibrillation using TEM and Thioflavin T fluorescence assay. In the absence of additives, lysozyme showed extensive fibrillation; however, upon addition of nanogels, the formation of toxic fibrils was markedly suppressed (Fig. 4A). As also observed in the enzymatic activity assay, measurements of Thioflavin T fluorescence showed that an increase in molecular weight and hydrophobicity resulted in greater suppression of fibril formation (Fig. 4B). The same trend was observed when the nanogel concentration was increased (Figure S5). In the presence of NG-F, less than 10% fibrillation is observed even after heating at high temperatures for 30 minutes, a value that is much lower than that obtained with linear polymers (poly-SPB) and previously reported compounds such as non-detergent sulfobetaines^33^,^34^,^35^,^36^,^37^ and L-arginine hydrochloride^38^,^39^ (Figure S6).

### Nanogels preserve higher-order structures of lysozyme

**Figure 5:**
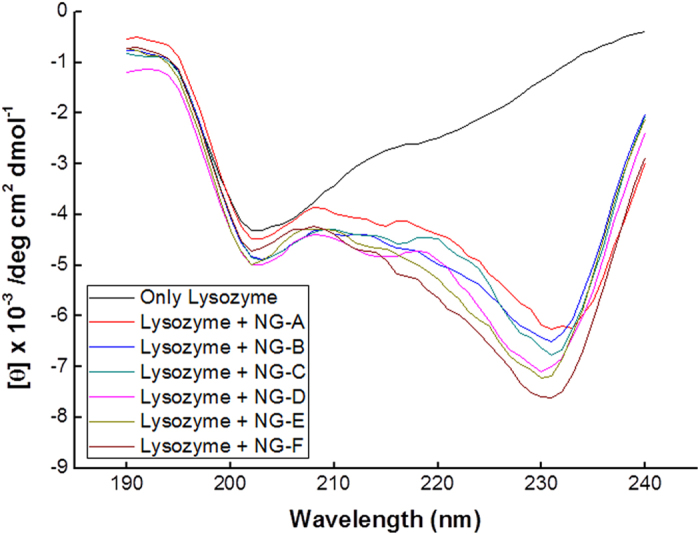
Representative far-UV CD spectra of lysozyme in the presence of various nanogels (2% polymer concentration) after heating.

Next, the effect of nanogels on the higher-order structures of lysozyme was studied by circular dichroism (CD) spectroscopy. In the absence of additives, a remarkable decrease in the intensity of the CD bands of lysozyme was observed upon heating (Figure S7). However, when nanogels were added to the lysozyme solution, the reduction in intensity of the CD bands due to heating was much lower, as clearly shown in Fig. 5. This suggests that higher-order structures were partially preserved in the presence of nanogels. Moreover, an increase in hydrophobicity and molecular weight resulted in greater retention of secondary structures. For further confirmation, the secondary structure elements were estimated by deconvolution of the CD spectra using the CDSSTR algorithm with reference set 7 of the Dichroweb server^40^,^41^, and the results are summarized in Table 2. Previous studies have reported that fibrillar assemblies are very stable and are primarily composed of β-sheets in a characteristic cross-β conformation^42^. As expected, the β-sheet content of lysozyme alone increased from 18% to 34% upon heating, at the expense of the α-helix content, which decreased from 57% to 3%. However, in the presence of nanogels, the β-sheet content of lysozyme was markedly lower, and the α-helix and turn contents also remained constant before and after heating. This suggests that nanogels promoted the partial retention of the higher-order structures of lysozyme.

### Conformational states of lysozyme

**Figure 6:**
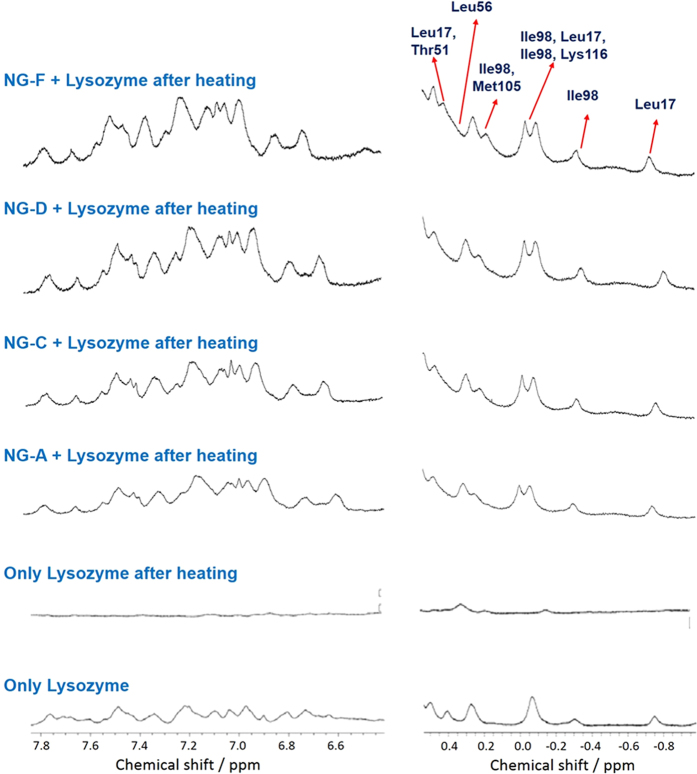
^1^H NMR spectra of a mixture of lysozyme with and without additives, before and after heating at 90 °C for 30 min.

It is well known that folded proteins, or proteins in their native state, shows an extensive range of chemical shift in NMR studies, owing to the anisotropic magnetic fields of the aromatic or carbonyl groups. In contrast, aggregated or denatured proteins tends to unfold from their native state and transforms into random-coil conformation, thus they show relatively narrow range of chemical shifts^43^. From Fig. 6, it is clearly evident that when lysozyme is heated in the absence of any additive, lysozyme signals corresponding to different amino acid residues disappeared. This suggests that lysozyme transforms into random coil conformation and thus loses its secondary structure on heating at such elevated temperatures. All the lysozyme peaks can be assigned to their corresponding residues, based on previous literatures^44^,^45^. When nanogels were added to lysozyme prior to heating, almost all the lysozyme signals in both upfield and download regions were still present, indicating that lysozyme remained dissolved in the solution and also retained partial higher order structures^46^, indicating that nanogels intercepted the transformation of lysozyme into random coil conformation and unfolding. Furthermore, the signals denoted in Fig. 6 represent the residues located in or near the secondary structure elements (α-helix, β-sheet and loop) and the retention of all these signals clearly shows that nanogels stabilizes the lysozyme even under extreme heating, thus preserving the native state of lysozyme.

Proteins tends to completely or partially misfold under certain conditions which exposes hydrophobic domains of the proteins. Intermolecular interactions between such domains of structural subunits induce protein aggregation^47^. As discussed in our previous report^10^, polymers can suppress protein aggregation: because of their weak and reversible interaction with proteins, they act as molecular shields, reducing collisions between aggregating species and maintaining the water structure^29^. The improved efficiency of nanogels as compared to linear polymers can be attributed to the tendency of nanogels to act as artificial molecular chaperones, by trapping proteins and protecting them against extreme conditions such as heating, drying, and desiccation.

## Conclusion

In summary, we have shown that nanogels based on zwitterionic polymers suppress the thermal aggregation of proteins with high efficiency. To our knowledge, this is the first report of zwitterionic polymer-based nanogels for the inhibition of protein aggregation. The nanogels exhibited a higher efficiency than the linear analogues and many previously reported compounds. Upon heating in the presence of nanogels, lysozyme retained high enzymatic activity and its higher-order structures, and amyloid fibril formation was suppressed. A more detailed study is necessary to gain a better and reliable understanding of nanogels, which would enable their application in the development of protein biopharmaceuticals. Current efforts are underway to optimize the efficiency of nanogels by modifying various parameters of the core and shell, as well as to elucidate the mechanism of protection.

## Methods

### Materials

Sulfobetaine (SPB) monomer was donated by Osaka Organic Chemical (Osaka, Japan) and used without further purification. 2-(Dodecylthiocarbonothioylthio)-2-methylpropionic acid, Thioflavin T (ThT), *Micrococcus lysodeikticus*, and lysozyme from chicken egg white were purchased from Sigma-Aldrich. Azobisisobutyronitrile (AIBN) was purchased from Wako Pure Chemical Industries (Osaka, Japan) and was recrystallized from methanol before use. Ethylene glycol dimethacrylate, BuMA and 4,4′-azobis-(4-cyanovaleric acid) (V-501, initiator) were purchased from TCI (Tokyo, Japan). Prior to its use, the inhibitor from BuMA was removed by passing through an inhibitor removal prepacked column (Sigma-Aldrich).

### Synthesis of Poly-SPB

Polymers of different degrees of polymerization (DP) were synthesized by varying the monomer to RAFT agent ratio. As an example, the procedure for the synthesis of polymer with DP 200 is briefly described as follows: the SPB monomer (12 mmol), 2-(dodecylthiocarbonothioylthio)-2-methylpropionic acid (0.06 mmol), and AIBN (0.012 mmol) were dissolved in 60 mL methanol-water mixture (3:1, v/v). The solution was then purged with nitrogen gas for 1 h and stirred at 70 °C. After 6 h, the reaction mixture was dialyzed using a membrane of 14,000 MWCO (molecular weight cut-off) (Viskase Companies. Inc., Illinois, USA) successively against methanol and water for 24 h each with regular change of solvent. The polymer was obtained after lyophilisation.

### NMR Measurements

NMR measurement of the polymer was carried out on a 900 MHz Bruker Avance III HD spectrometer using D_2_O as the solvent (Figure S1). ^1^H-NMR measurements of lysozyme with and without nanogels were carried out on a 400 MHz Bruker Avance III spectrometer. All the data were processed using the Topspin 3.5 software.

### Molecular weight determination

The molecular weight and distribution (polydispersity index, PDI) of the polymers were determined by gel permeation chromatography (GPC, column, BioSep-s2000; Phenomenex, Inc., CA, USA), using a Shimadzu high-performance liquid chromatography data system incorporating a refractive index detector. A NaBr solution (pH 7.4, 0.1 M) was used as the mobile phase (flow rate: 1 mL min^−1^) and pullulan (Shodex Group, Tokyo, Japan) as the standard.

### Synthesis of core-shell nanogels

Nanogels were synthesized using polymers of different DP. As an example, the procedure for the synthesis of the nanogel with polymer of DP 200 is described as follows: poly-SPB (macro-CTA, 0.2 g), SPB (1.477 g), BuMA (44.4 μL), ethylene glycol dimethacrylate (21 μL), and V-501 (4 mg) were dissolved in 60 mL water-methanol mixture (5:1, v/v). The reaction mixture was purged with nitrogen gas for 1 h and stirred at 70 °C. After 24 h, the solution was dialyzed using a membrane of 50,000 MWCO (Spectra/Por^®^ Membrane, Spectrum Labs, USA) against water for 3 d with regular change of water. The final product was obtained by lyophilisation.

### Dynamic light scattering

The hydrodynamic diameter (HD) and size distribution of the nanogels at a concentration of 10 mg/mL were determined in a Zetasizer 3000 (Malvern Instruments, Worcestershire, UK) with a scattering angle of 173°.

### Transmission electron microscopy

The samples (nanogels and lysozyme after heating with and without nanogels) were dissolved in phosphate buffer saline (PBS; pH 7.4) and 10-μL aliquots of the samples were placed on a copper grid (NS-C15 Cu150P; Stem, Tokyo, Japan). The grids were negatively stained with 2% phosphotungstic acid (Sigma Aldrich, Steinheim, Germany) for 45 s, washed with two drops of distilled water, blotted, and air-dried.

### Residual enzyme activity

A lysozyme solution in PBS was mixed with a poly-SPB solution to achieve a final lysozyme concentration of 10 μM. The solution was heated at 90 °C for 30 min. Micrococcus lysodeikticus (2 mL; 0.25 mg/mL in PBS) was placed in a quartz cuvette and mixed with 100 μL lysozyme-polymer solution. The solution of intact cells of Micrococcus lysodeikticus is turbid, and its turbidity decreased upon degradation by lysozyme. The change in turbidity was measured by UV-Vis spectrophotometry (UV-1600PC, Shimadzu Corporation, Kyoto, Japan) at 600 nm from 0 to 6 min with constant stirring at room temperature. The rate of decrease in absorbance was calculated from the slope of the absorbance vs time plot, and this slope corresponds to the residual enzymatic activity.

### Thioflavin T assay

A stock solution of Thioflavin T (ThT) was prepared by adding 4 mg ThT to 5 mL PBS and filtered through a 0.22 μm filter. The stock solution was diluted by adding 1 mL of stock to 50 mL PBS to generate the working solution. The lysozyme solution in PBS was mixed with various reagents and heated to 90 °C for 30 min; then, 2 mL of this solution was mixed with 100 μL ThT and fluorescence was measured at an excitation wavelength of 450 nm and emission wavelength of 484 nm (JASCO FP-8600). The enhanced fluorescence of ThT is due to amyloid formation upon binding with amyloid fibrils.

### Circular dichroism spectroscopy

The far-UV circular dichroism (CD) spectra of lysozyme were used to monitor the changes in the secondary structure of lysozyme before and after heat treatment. The protein concentration and path length of the cell used were 10 μM and 0.5 cm, respectively, and the CD spectra were recorded on a JASCO-820 spectropolarimeter. The spectropolarimeter was purged with nitrogen gas before starting the experiments. Each spectrum was baseline-corrected and was collected as an average of three scans at a scan rate of 200 nm min^−1^ and a response time of 2 s.

### Statistical analysis

All data are expressed as mean ± standard deviation (SD). All experiments were conducted in triplicate. One-way analysis of variance with a post-hoc Fischer’s protected least significant difference test was used. Differences were considered statistically significant at a P value of <0.05.

## Additional Information

**How to cite this article:** Rajan, R. and Matsumura, K. Inhibition of protein aggregation by zwitterionic polymer-based core-shell nanogels. *Sci. Rep.* **7**, 45777; doi: 10.1038/srep45777 (2017).

**Publisher's note:** Springer Nature remains neutral with regard to jurisdictional claims in published maps and institutional affiliations.

## Supplementary Material

Supplementary Information

## Figures and Tables

**Table 1:** Characteristics of nanogels and Macro-CTAs nrepared via RAFT polymerization.

Nanogel	Macro-CTA	Molar ratio^a^	M_n_ × 10^−3b^	M_w_/M_n_^b^	% BuMA	Hydrodynamic diameter in nm^c^
NG-A	Poly-(SPB)_20_	100:1:5	5.5	1.18	0	14.38 ± 0.14 (0.40)^d^
NG-B					5	12.46 ± 0.35 (0.33)^d^
NG-C					10	11.04 ± 0.19 (0.22) ^d^
NG-D	Poly-(SPB)_200_	1000:1:5	36.2	1.59	0	24.52 ± 0.58 (0.41)^d^
NG-E					5	23.68 ± 0.64 (0.26)^d^
NG-F					10	20.0 ± 0.72 (0.51)^d^

^a^[monomer]:[initiator]:[RAFT agent]. ^b^Determined by GPC. ^c^Determined by DLS. ^d^Polydispersity index, determined by DLS.

**Table 2:** Estimates of the secondary structural components of lysozyme in the absence and presence of nanogels (2% concentration) as obtained from CD spectral analyses.

Time	Secondary structure content	Only Lysozyme	NG-A	NG-B	NG-C	NG-D	NG-E	NG-F
Before heating	α-helix	57%	41%	42%	44%	42%	44%	43%
	β-Sheet	18%	19%	19%	19%	18%	19%	20%
	Turn	8%	14%	12%	13%	14%	10%	12%
	Unordered	16%	26%	27%	25%	20%	27%	26%
After heating	α-helix	3%	23%	23%	24%	25%	25%	26%
	β-Sheet	34%	21%	20%	19%	19%	18%	18%
	Turn	21%	17%	19%	17%	15%	17%	16%
	Unordered	40%	38%	38%	39%	42%	40%	39%
