# Supplementary material for: Inhibition of protein aggregation by zwitterionic polymer-based core-shell nanogels
Source: Sci Rep. 2017 Apr 4;7:45777. doi: 10.1038/srep45777 (PMC5379557; doi:10.1038/srep45777)
Supplement: Supplementary Information [file srep45777-s1.pdf]

Supplementary Information

Inhibition of protein aggregation by zwitterionic polymer-based core-shell nanogels

Robin Rajan and Kazuaki Matsumura

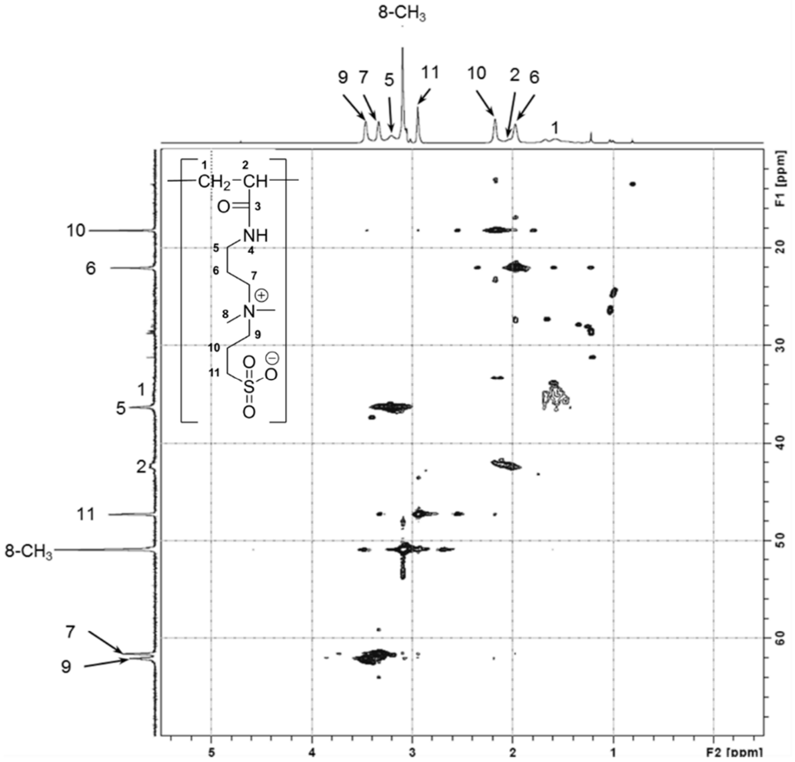

Figure S1. 2D NMR spectra of poly-SPB (D_2O, 900 MHz).

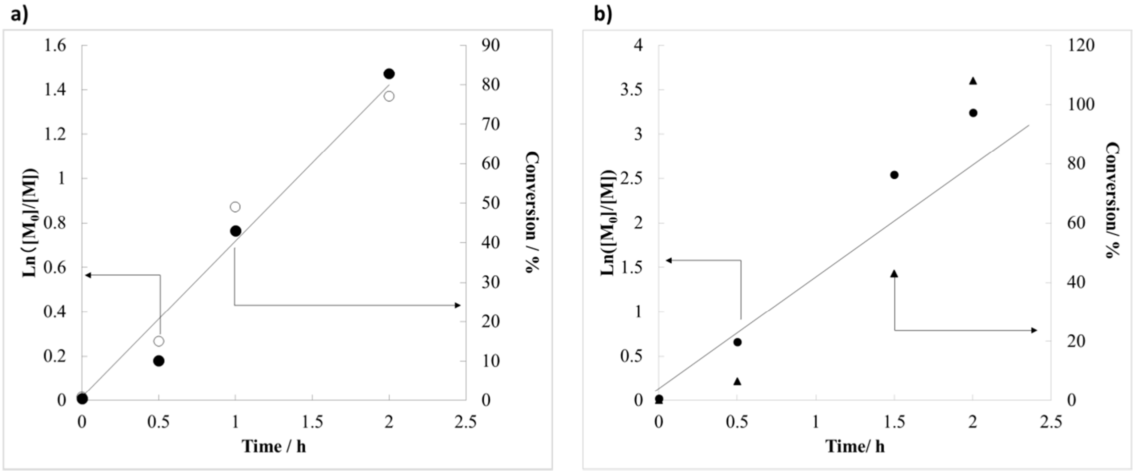

Figure S2. Kinetic plot of poly-SPB with a) DP 20 and b) DP 200.

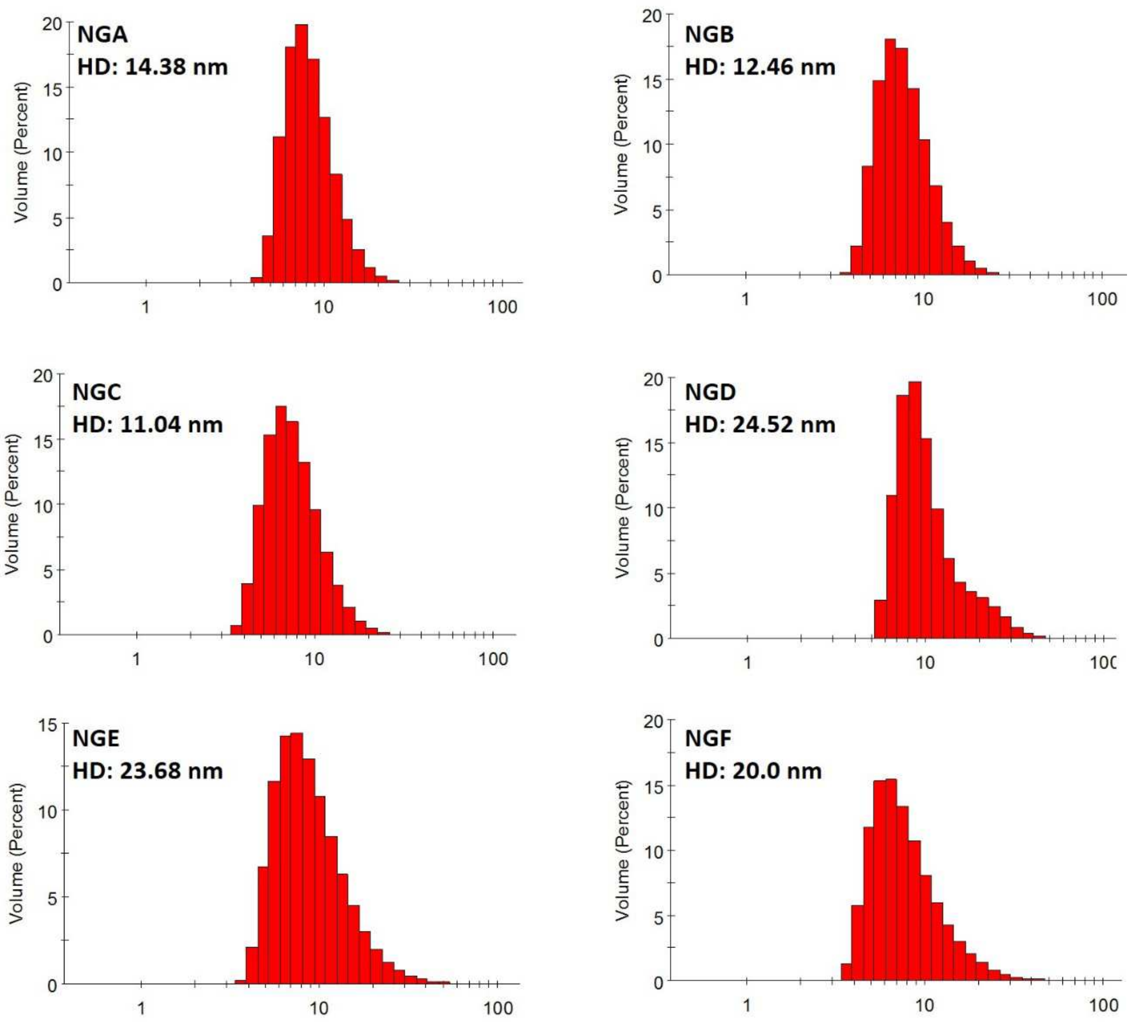

Figure S3. Size distribution by volume of nanogels, measured at a concentration of 10 mg/mL. The corresponding HD is shown next to each distribution plot.

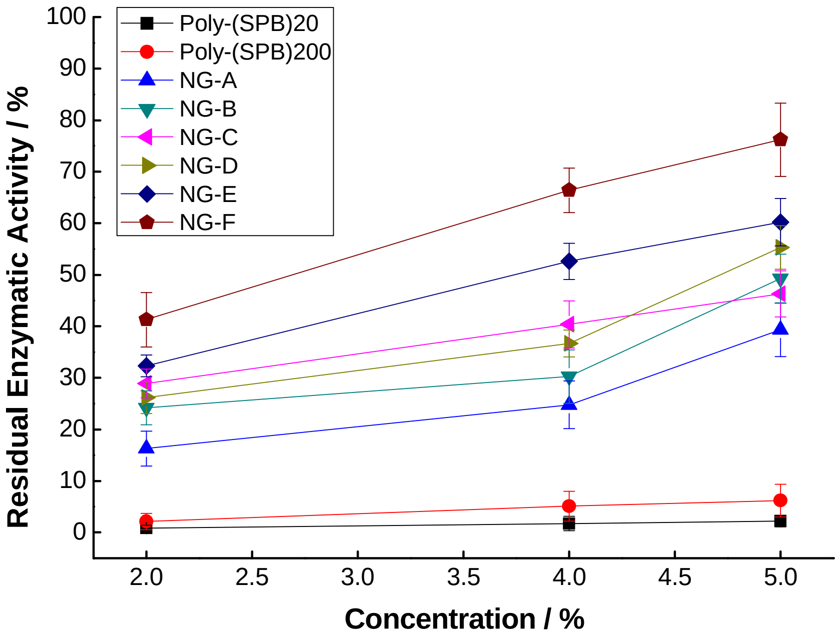

Figure S4. Enzymatic activity of lysozyme after treatment at 90 °C in the presence of different nanogels at various concentrations (% w/v). Data are expressed as the mean ± SD of three independent experiments.

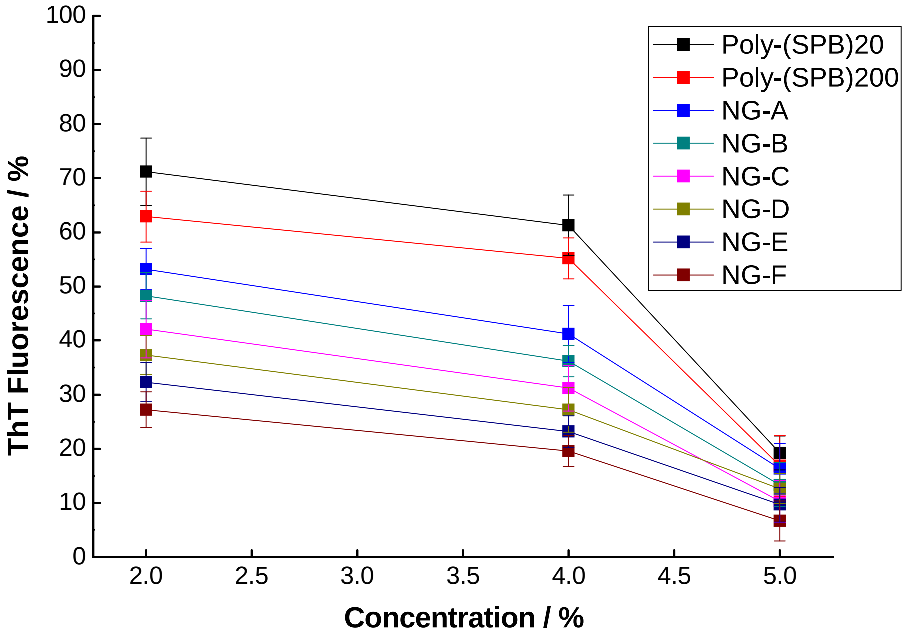

Figure S5. Thioflavin T fluorescence of lysozyme upon heating to 90 °C for 30 min in the presence of different nanogels at various concentrations (% w/v). Data are expressed as the mean ± SD of three independent experiments.

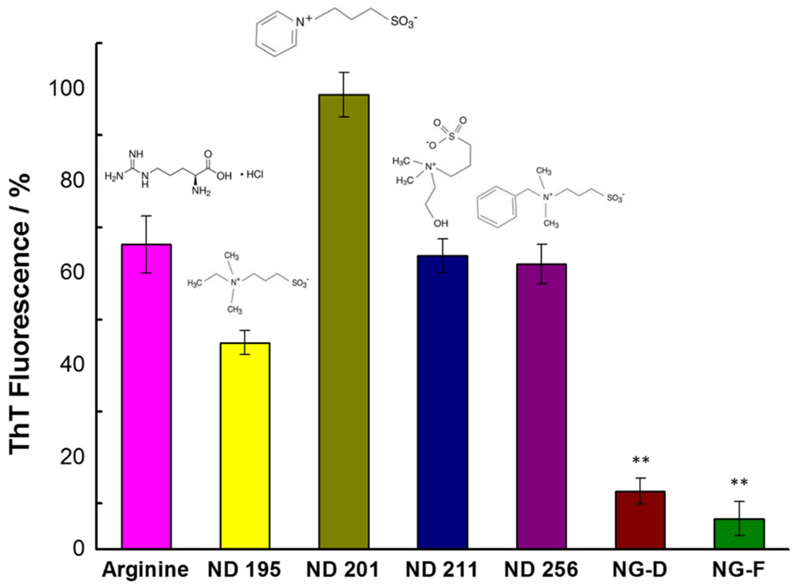

Figure S6. Protein aggregation of lysozyme upon heating to 90 °C for 30 min in the presence of various reagents (5% w/v). Data are expressed as the mean ± SD of three independent experiments. $^{**}p < 0.01$ vs. Arginine, ND195, NF201, ND211, ND256.

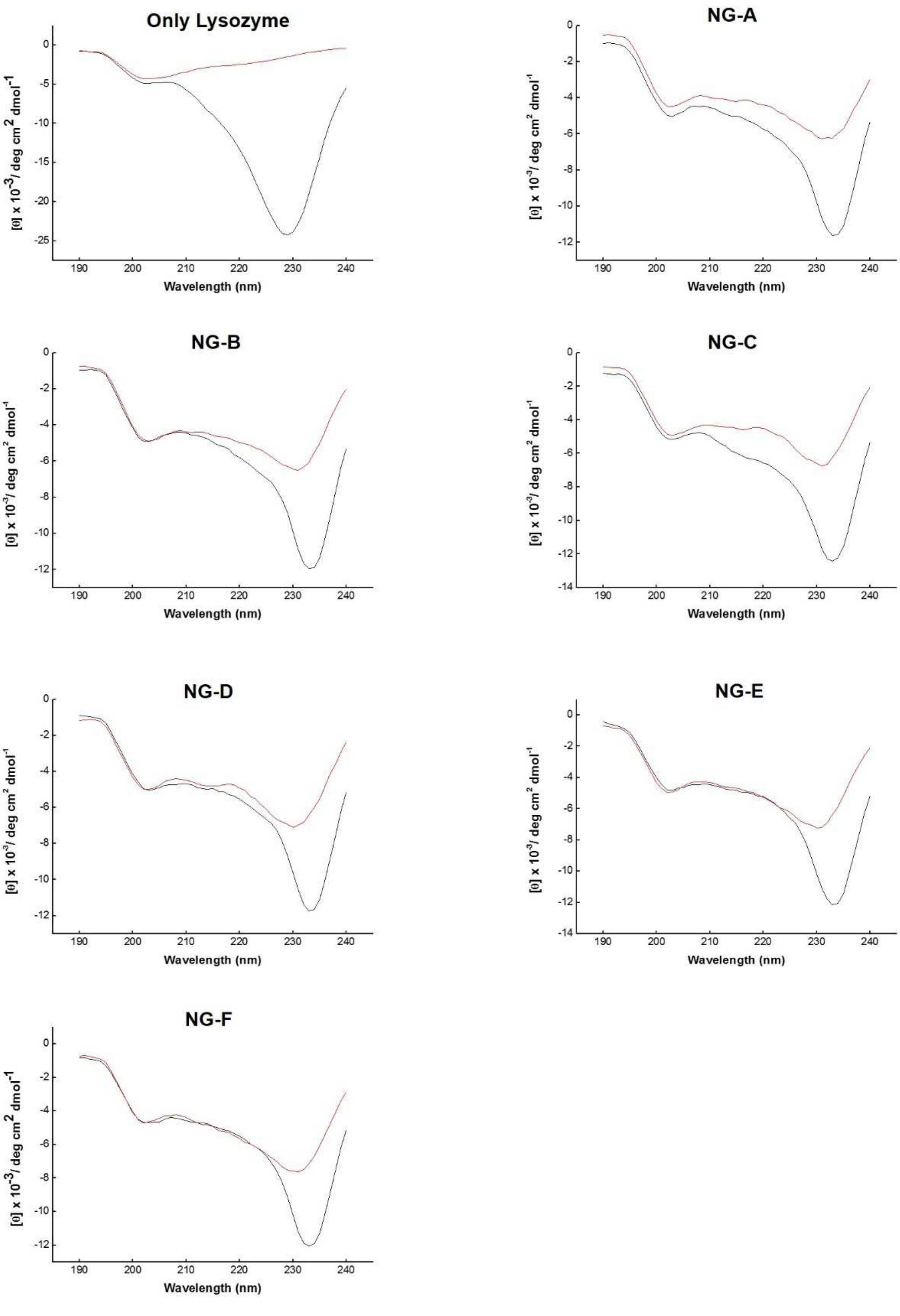

Figure S7. Representative far-UV CD spectra of lysozyme in the presence of various nanogels (2% polymer concentration) before (black line) and after heating (red line).
